# Reply to Polizzi and Dowd: Within-country counterfactual reveals importance of retirement age mortality in addition to established concern about working ages

**DOI:** 10.1073/pnas.2320028121

**Published:** 2024-01-16

**Authors:** Leah R. Abrams, Mikko Myrskylä, Neil K. Mehta

**Affiliations:** ^a^Department of Community Health, Tufts University, Medford, MA 02155; ^b^Max Planck Institute for Demographic Research, Rostock 18057, Germany; ^c^Population Research Unit, University of Helsinki, Helsinki 00014, Finland; ^d^Max Planck–University of Helsinki Center for Social Inequalities in Population Health, Rostock 18057, Germany; ^e^Department of Epidemiology, University of Texas Medical Branch, Galveston, TX 77555

Polizzi and Dowd (2023) show that working age mortality has more prominent effects on recent US life expectancy (LE) trends when applying the rates of change of other countries to the United States (1). This finding is broadly consistent with many existing studies that have found that the United States is experiencing uniquely troubling mortality patterns at working ages compared to peer nations (2–7). These cross-country comparisons are part of a larger conversation among researchers and policy makers about the health and well-being of the US working age population (8).

While the counterfactual of Polizzi and Dowd asks what US LE at age 25 would be if the United States had the mortality trends of peer nations, ours asks what U.S. LE at age 25 would be if sex- and age-specific mortality rates had continued their trends from the prior decade (9). Each counterfactual addresses substantively different questions, and consequently the answers will also differ. Our analysis is about why U.S. LE improvements slowed down compared to its own past performance, not about why U.S. mortality trends differ from those of other countries. For our research question, the relevant comparison is a country’s own history. We argue that within-country counterfactuals are critically important for answering the question of how to return to a prior rate of LE improvement and provide insights into the mechanisms that might underlie shifts in mortality trends in this population.

Polizzi and Dowd (2023) argue that our counterfactual uses a decade when working age mortality was already stagnating. U.S. LE at age 25 improved by 1.54 y in that decade (2000 to 2009), a rate similar to the average of the prior three decades, compared to 0.22 y in 2010 to 2019 (10). Because LE trends significantly changed around 2010, we looked to mortality conditions in the decade prior when LE growth was robust. In 2000 to 2009, mortality at ages 25 to 64 declined modestly before increasing in 2010 to 2019. Retirement age mortality declined substantially during 2000 to 2009 and then declines slowed in 2010 to 2019. Therefore, mortality trends in both age groups became more adverse. Our analysis indicates that trends in retirement ages had larger effects than trends in working ages, even when considering metrics that more heavily weight a loss of life at younger ages, such as years of life lost.

Our study highlights the contribution of working age mortality trends to LE trends, while also identifying another underappreciated factor—slowing mortality improvements at retirement ages. The double jeopardy of both age groups emerges clearly when comparing the United States to itself in the earlier period.
